# Predicting Protein Function from Structure—The Roles of Short-chain Dehydrogenase/Reductase Enzymes in *Bordetella* O-antigen Biosynthesis

**DOI:** 10.1016/j.jmb.2007.09.055

**Published:** 2007-11-30

**Authors:** Jerry D. King, Nicholas J. Harmer, Andrew Preston, Colin M. Palmer, Martin Rejzek, Robert A. Field, Tom L. Blundell, Duncan J. Maskell

**Affiliations:** 1 Department of Veterinary Medicine, Madingley Road, University of Cambridge, Cambridge CB3 0ES, UK; 2 Department of Biochemistry, 80 Tennis Court Road, University of Cambridge, Cambridge CB2 1GA, UK; 3 Department of Molecular and Cellular Biology, University of Guelph, Ontario, Canada N1G 2W1; 4 School of Chemical Sciences and Pharmacy, University of East Anglia, Norwich NR4 7TJ, UK

**Keywords:** dTDP, deoxythymidine diphosphate, l-GalNAc3NAcA, 2,3-diacetamido-2,3-dideoxy-l-galacturonic acid, GME, GDP-mannose 3,5-epimerase, GMER, GDP-4-keto-6-deoxymannose 3,5-epimerase/reductase, LPS, lipopolysaccharide, PDB, Protein Data Bank, BLAST, basic local alignment search tool, SDR, short-chain dehydrogenase/reductase, UDP-d-ManNAc3NAcA, UDP-2,3-diacetamido-2,3-dideoxy-d-mannuronic acid, UDP-l-GalNAc3NAcA, UDP-2,3-diacetamido-2,3-dideoxy-l-galacturonic acid, UMP, uridine monophosphate, MCS, multiple cloning site, short-chain dehydrogenase/reductase, X-ray crystallography, *Bordetella*, lipopolysaccharide, O-antigen biosynthesis

## Abstract

The pathogenic bacteria *Bordetella parapertussis* and *Bordetella bronchiseptica* express a lipopolysaccharide O antigen containing a polymer of 2,3-diacetamido-2,3-dideoxy-l-galacturonic acid. The O-antigen cluster contains three neighbouring genes that encode proteins belonging to the short-chain dehydrogenase/reductase (SDR) family, *wbmF*, *wbmG* and *wbmH*, and we aimed to elucidate their individual functions. Mutation and complementation implicate each gene in O-antigen expression but, as their putative sugar nucleotide substrates are not currently available, biochemical characterisation of WbmF, WbmG and WbmH is impractical at the present time. SDR family members catalyse a wide range of chemical reactions including oxidation, reduction and epimerisation. Because they typically share low sequence conservation, however, catalytic function cannot be predicted from sequence analysis alone. In this context, structural characterisation of the native proteins, co-crystals and small-molecule soaks enables differentiation of the functions of WbmF, WbmG and WbmH. These proteins exhibit typical SDR architecture and coordinate NAD. In the substrate-binding domain, all three enzymes bind uridyl nucleotides. WbmG contains a typical SDR catalytic TYK triad, which is required for oxidoreductase function, but the active site is devoid of additional acid–base functionality. Similarly, WbmH possesses a TYK triad, but an otherwise feature-poor active site. Consequently, 3,5-epimerase function can probably be ruled out for these enzymes. The WbmF active site contains conserved 3,5-epimerase features, namely, a positionally conserved cysteine (Cys133) and basic side chain (His90 or Asn213), but lacks the serine/threonine component of the SDR triad and therefore may not act as an oxidoreductase. The data suggest a pathway for synthesis of the O-antigen precursor UDP-2,3-diacetamido-2,3-dideoxy-l-galacturonic acid and illustrate the usefulness of structural data in predicting protein function.

## Introduction

The acceleration of genome sequencing has created a challenge for modern biology, namely, interpretation of the vast and growing amount of genetic information in the databases. Search algorithms such as the basic local alignment search tool (BLAST)^1^ can efficiently find related genes, but only in a small minority of cases is the precise function of a novel enzyme equivalent to that of the highest scoring hit. In straightforward cases the output is useful to generate hypotheses for the novel enzyme's function, based on common catalytic chemistry, substrates or both, and these postulates are then amenable to direct biochemical investigation. To make full use of genome data it is important that where such standard bioinformatic analysis cannot predict precise roles and biochemical function assays are not possible, tools are developed to differentiate the functions of genes. A good example of such a challenging case is where gene products are annotated as short-chain dehydrogenase/reductases (SDRs). Members of this family share low sequence identity (typically 15–30%) and catalyse a wide range of chemical reactions, including oxidation, reduction, epimerisation, dehydration and decarboxylation (reviewed in Ref. 2).

*Bordetella* *parapertussis* and *Bordetella bronchiseptica* are pathogens of mammals. *B. bronchiseptica* is associated with respiratory tract infections in many animals including acute tracheobronchitis in dogs (kennel cough)^3^ and atrophic rhinitis in pigs,^4^ whereas *B. parapertussis* can cause whooping cough in humans.^5^ A separate lineage of *B. parapertussis* infects sheep.^6^

The major component of the outer leaflet of the outer membrane in Gram-negative bacteria is lipopolysaccharide (LPS). The complete structure of this macromolecule in *B. bronchiseptica* and *B. parapertussis* has been recently described.^7^ The lipid A domain of LPS, which consists of a diglucosamine backbone substituted with fatty acyl chains, forms the outer leaflet of the outer membranes of the bacteria. Lipid A is linked to a complex, branching oligosaccharide known as the core. Lipid A-core comprises a proportion of the LPS that is exposed on the cell surface and is known as band B. In *B. bronchiseptica*, lipid A-core can be substituted with a trisaccharide and this structure is known as band A. Synthesis of the band A trisaccharide requires functions encoded in the *wlb* gene cluster (previously named *bpl*).^8^

Both *B. bronchiseptica* and *B. parapertussis* also substitute their LPS with an O antigen. This O antigen contains 12 to 15 2,3-diacetamido-2,3-dideoxy-l-galactosaminuronic acid (l-GalNAc3NAcA) residues^9^ and is required for full virulence in animal and *in vitro* models of infection.^10^ O-antigen biosynthesis requires genes in the *wbm* cluster that is adjacent to the *wlb* genetic locus. The *wbm* locus contains three neighbouring SDR genes (*wbmF*, *wbmG* and *wbmH*), all of which have been annotated as nucleotide-sugar epimerases/dehydratases.

The structure of complete O antigen and the homology of *wbm* genes with genes of known function have led us to propose a pathway for biosynthesis of this polysaccharide and we are currently testing various steps in this scheme as part of an ongoing project to determine the functions of all 24 *wbm* genes. This report specifically concerns the roles of the SDR genes *wbmF*, *wbmG* and *wbmH*.

O-antigen residues are synthesised as sugar nucleotide precursors. The probable substrate for *wbm* locus-encoded biosynthesis of the nucleotide-activated l-GalNAc3NAcA is UDP-2,3-diacetamido-2,3-dideoxy-d-mannuronic acid (UDP-d-ManNAc3NAcA). This compound is related to the l-*galacto* configuration by inversion of the stereochemistry at the hexose 3 and 5 positions. In the bordetellae, UDP-d-ManNAc3NAcA is formed as a precursor for band A trisaccharide synthesis by 2-epimerisation of UDP-2,3-diacetamido-2,3-dideoxy-d-glucuronic acid, catalysed by WlbD.^11^ Because there are precedents for either a single^12^ or multiple^13–16^ SDR enzymes catalysing 3,5-epimerisation conversions of sugar nucleotides, we hypothesise that one or more of WbmF, WbmG and WbmH operate in this biosynthetic pathway to catalyse the conversion of UDP-d-ManNAc3NAcA to UDP-2,3-diacetamido-2,3-dideoxy-l-galacturonic acid (UDP-l-GalNAc3NAcA). Because of the low percentage conversion of the WlbD-catalysed reaction, UDP-d-ManNAc3NAcA is not currently available for biochemical studies and therefore this hypothesis cannot be directly tested at the present time.

In this report we demonstrate the involvement of *wbmF*, *wbmG* and *wbmH* in O-antigen expression by mutation of the genes in *B. bronchiseptica*. Because the putative substrate is unavailable, we elected to probe the catalytic functions of WbmF, WbmG and WbmH by characterising these proteins' three-dimensional structures. With soaking and co-crystallisation experiments, densities corresponding to cofactors and nucleotide portions of substrate analogues can be identified within the active sites. Biochemical and crystallographic studies have defined the structural basis for the catalytic chemistries of a range of other SDR enzymes (reviewed in Ref. 17). Our knowledge of these structure–function relationships enables us to interpret the structures of the *wbm* SDRs. We have analysed the potential for acid–base chemistry in the active sites to differentiate their potential roles *in vivo*. Based on the composition of O antigen, the genetic context of these *wbm* genes and information from the structural studies, we propose roles for these genes in O-antigen biosynthesis. The results demonstrate the usefulness and limitations of X-ray data in elucidating biochemical pathways where catalytic activity cannot be directly measured.

## Results

### Mutational analysis of *wbmF*, *wbmG* and *wbmH*

**Fig. 1:**
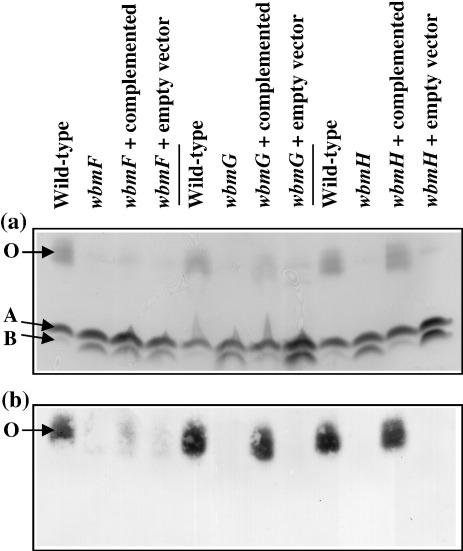
(a) Silver stain analysis and (b) Western immunoblot of duplicate SDS-PAGE gels showing the LPS profiles of wild-type *B. bronchiseptica* (CN7635E) and CN7635E-derived mutants in *wbmF* (CNF0a), *wbmG* (CNG1a) and *wbmH* (CNH1d) and mutants carrying the complementation vectors for *wbmF* (pCompF), *wbmG* (pCompG) and *wbmH* (pCompH) or the empty vector (pCompEmpty). The positions of the *B. bronchiseptica* A-band (A) and B-band (B) species are indicated as well as the position of LPS that contains O antigen (O). The primary monoclonal antibody used in (b) that recognises O-band LPS was D13B11.^35^

To test the hypothesis that *wbmF*, *wbmG* and *wbmH* are involved in O-chain biosynthesis, each gene was disrupted by insertion of a tetracycline-resistance cassette. Mutation of the chromosomal genes was confirmed by Southern hybridisation (data not shown). The effects of these mutations were assessed by silver-stained SDS-PAGE analysis and immunoblotting of mutant LPS (Fig. 1). Mutation of *wbmG* or *wbmH* results in apparent abrogation of O-antigen synthesis, as the LPS from these mutants lacks O band as detected by either silver stain or Western blot. O-antigen expression in the *wbmF* mutant, CNF0a, is dramatically reduced compared with the wild type, but this strain retains its ability to express a small amount of material with the electrophoretic mobility of O-band LPS and which binds the O-antigen-specific monoclonal antibody, D13B11.

Each of the mutations was complemented by expression of the cognate wild-type allele *in trans* (Fig. 1). Each coding sequence was cloned into the broad host range vector pBBR1MCS(kan) under the control of the *flaA* promoter to generate complementation vectors. Complementation of the *wbmG* and *wbmH* mutations restored O-antigen expression to near wild-type levels. O-antigen expression in the complemented *wbmF* mutant was increased relative to the *wbmF* mutant, but was still considerably less than in wild type. The reason for incomplete complementation of the *wbmF* mutation is unknown, but is not likely to be due to polar effects of the mutation because, due to the organisation of genes in the *wbm* locus, *wbmF* is at the 3′ end of an operon.^18^ Introduction of the empty complementation vector had no effect on O-antigen expression in any of the mutants.

### The gene products WbmF, WbmG and WbmH are all in the extended SDR family

**Fig. 2:**
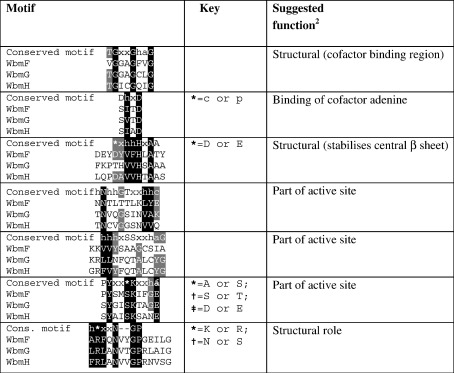
Conserved motifs in extended SDR enzymes. Sections from Wbm SDR protein sequences are aligned with the characteristic motifs of the extended SDR subfamily. Except for the alignments with Wbm sequences, the information in this table is from Ref. 2. Lowercase letters represent conserved biophysical properties of amino acid side chains: x, any residue; h, hydrophobic; c, charged; p, polar; a, aromatic. Conservation of motif features in Wbm proteins is indicated by shading.

The SDR family is divided into the classical and extended subfamilies primarily on the basis of protein length and the sequence pattern of the glycine-rich cofactor-binding region close to the N terminus. According to both criteria, the products of *wbmF*, *wbmG* and *wbmH*, which are predicted to have 357, 310 and 313 amino acids, respectively, and all have the GxxGxxG motif near the N terminus, are extended SDRs^2^ (Fig. 2).

WbmG and WbmH are closely related to each other, sharing 38% amino acid identity. WbmF, WbmG and WbmH are all homologous to Pfam01370 (NAD-dependent epimerase/dehydratase family). The most closely related, characterised homologues of these Wbm SDRs in the protein databases are deoxythymidine diphosphate (dTDP)-glucose 4,6-dehydratases from *Escherichia coli* and *Streptomyces venezuelae* with 27% or 28% sequence identity (Table 1).^19^ This level of sequence conservation between members of the SDR family does not imply conservation of either catalytic chemistry or substrate; rather this functional information can be better determined by examination of the residues at key positions in the active sites and binding pockets. Several conserved motifs within the extended SDR subfamily facilitate alignment of sections of the Wbm SDR sequences with homologues (Fig. 2) but many of the critical functional amino acids are located outside of these motifs, and with this low level of conservation, it is not possible to make full sequence alignments with confidence that all of these residues are correctly identified.

### Crystallisation of His_6_-WbmF, His_6_-WbmG and His_6_-WbmH

Thus far the data implicated each of these *wbm* SDRs in O-antigen synthesis, but did not indicate precise roles for each gene. Therefore, in order to elucidate their individual functions we characterised the gene products by X-ray crystallography. The overexpression, purification and crystallisation of these proteins are described elsewhere.^20^ The structures discussed in the present work are summarised in Table 2.

### General architecture of His_6_-WbmF, His_6_-WbmG and His_6_-WbmH

**Fig. 3:**
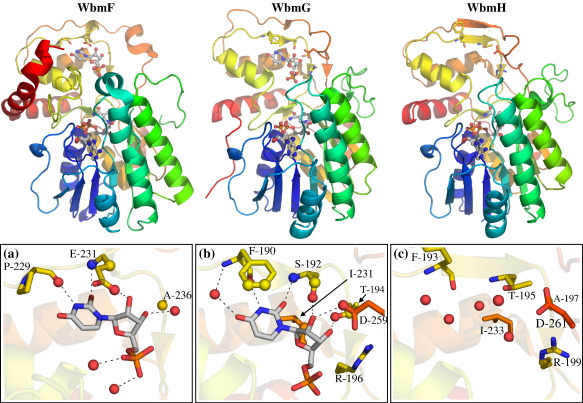
Nucleotide binding in WbmF, WbmG and WbmH. Proteins are oriented with the Rossmann domains in the lower part of each structure. Cartoons are used to represent protein backbone; bound nucleotides are shown as ball-and-stick and key protein side chains as sticks. The boxed panels show details of UMP-binding pockets in (a) His_6_-WbmF, UDP co-crystal, (b) His_6_-WbmG crystal soaked with UDP-glucose and (c) His_6_-WbmH. In (a) and (b), UMP is shown as sticks, and spheres represent atoms within 3.5 Å of the bound nucleotide. Probable hydrogen-bonding interactions are shown as dashed lines. In (c) spheres represent ordered water molecules in this binding pocket. Carbon atoms are coloured rainbow for protein, white for bound nucleotides; oxygen atoms are coloured red; nitrogen is blue and phosphorus orange.

These proteins all exhibit the typical SDR family architecture (Fig. 3). The structures each comprise two domains. The first of these is the N-terminal Rossmann fold domain in which a central β sheet is flanked by two layers of α helices. The cofactor-binding motif GxxGxxG is located at the C-terminal edge of this β sheet, which has seven parallel β strands running in the order 3, 2, 1, 4, 5, 6, 7. The second domain is largely made up of C-terminal sequence and contains all of the residues involved in binding of the nucleotide portion of the substrate. The catalytic sites in SDR enzymes are located at the interface of these two domains where, in sugar-nucleotide-modifying enzymes, the substrate hexose is brought into proximity with the cofactor nicotinamide ring. The main architectural differences between these three proteins lie in their C-terminal regions (Fig. 3). The C terminus of His_6_-WbmG consists of a loop that stretches out from the C-terminal domain to interact with the Rossmann domain. In His_6_-WbmH, this loop is not visible in the density, although this difference may not reflect any distinction between the real structures of the two proteins in solution. In WbmF, the last 30 residues form a large bent helix that covers two faces of the C-terminal domain. There is also an insertion of 14 amino acids in WbmF relative to the other structures, including all residues from Gly198 to Arg212. This extra loop extends over the nicotinamide end of the cofactor-binding pocket.

SDRs typically form dimers or higher oligomers. The crystal packing of these proteins suggests that WbmF, WbmG and WbmH have the conserved four-helix bundle usually found at the dimer interface. Analytical gel filtration chromatography indicated a hydrodynamic radius for each of these proteins consistent with either a monomer or dimer (data not shown). We conclude, therefore, that these proteins are present in solution predominantly in dimer form. In the WbmG and WbmH structures, the dimer interaction buries 1316 Å^2^ (5.7% of total protein surface) and 1562 Å^2^ (7.2%) of surface per dimer, respectively. In the WbmF structure the interface region is extended to include a short antiparallel β sheet composed of the residues Ile151 to Ser153 from each monomer as well as the loops that connect these strands to the bundle (Leu154 to Pro160); 2131 Å^2^ is buried in the WbmF dimer interface, which is equivalent to 8.6% of the total protein surface. In all three cases, the majority of the residues buried at these interfaces are hydrophobic or aromatic.

### Cofactor binding

NAD can be modelled into the electron density in all three proteins. His_6_-WbmG and His_6_-WbmH copurify with this compound from the *E. coli* expression host, but in order to stabilise the protein in solution His_6_-WbmF required the addition of exogenous cofactor,^20^ and this is the most likely source of this molecule in these His_6_-WbmF structures. The preference in each case for NAD cofactor as opposed to NADP is indicated by the close interaction (2.7 Å hydrogen bonds) of an aspartate side chain with the 2′-hydroxyl of the adenylic ribose. This side chain would disfavour binding of NADP by both steric and electrostatic repulsion of the NADP 2′-phosphate. In some SDR structures [e.g., GDP-4-keto-6-deoxymannose 3,5-epimerase/reductase (GMER)^21^ and RmlD^22^] the bound cofactor has direct access to solvent, indicating a pathway *via* which spent NAD/NADP can be replaced from solution. In these structures, the bound cofactor is buried within the core of the protein; in the case of the His_6_-WbmF structure, the 14-amino-acid insertion supplies an additional occluding loop, further blocking release of cofactor in this conformation.

### Binding of substrate

Soaking and co-crystallisation experiments with His_6_-WbmF and His_6_-WbmG revealed the binding mode for the nucleotide portion of the sugar nucleotide substrates of these enzymes (Fig. 3). The X-ray data from a His_6_-WbmG crystal soaked with UDP-glucose revealed extra density that could be modelled as far as the α-phosphate. The location of this molecule is consistent with the nucleotide-binding site for SDR homologue structures and with the hydrogen-bonding chemistry expected to bind uridine. No density was visible corresponding to the hexose portion of the soaked compound nor the second phosphate, suggesting that either this portion of the molecule is disordered within the crystal, or the compound was partially hydrolysed and the smaller uridine monophosphate (UMP) preferentially soaked into the substrate-binding pocket. In either case, the data indicate that the glucose-1-phosphate moiety of UDP-glucose is not strongly bound in a single conformation by WbmG. A similar soaking experiment but with GDP-mannose did not indicate any binding of this sugar nucleotide (data not shown), indicating a possible preference for the uracil base. The hydrogen bonding between the WbmG pocket and UMP suggests a basis for this preference (Fig. 3b). Although we were unable to obtain any complex of WbmH with a substrate analogue bound, the nucleotide-binding domain of WbmH is conserved in sequence and structure with WbmG, implying identical specificity and a common binding mode for the nucleoside portion of their substrates (Fig. 3c). UDP density to the α-phosphate is also visible in the WbmF/NAD/UDP ternary complex. The UMP density in this structure and the chemistry of its interaction with the protein suggests that this protein, like WbmG and WbmH, recognises a UDP-sugar substrate.

**Fig. 4:**
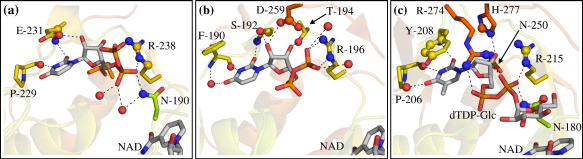
The UDP-binding pockets are shown for (a) His_6_-WbmF, soaked with UDP and (b) the His_6_-WbmG, UDP co-crystal. Spheres represent atoms within 3.5 Å of the bound UDP; NAD indicates the nicotinamide ring of the NAD cofactor. (c) dTDP-glucose bound in the active site of a D128N, E129Q mutant of dTDP-glucose 4,6-dehydratase DesIV from *S. venezuelae* (PDB ID 1R6D).^23^ The substrate-binding pockets are all shown from the same angle to enable comparison of the relative positions of the UDP diphosphates in (a) and (b) with the phosphates in dTDP-glucose in (c).

Both α and β nucleotide phosphates could be modelled in the data from a UDP soak into a WbmF-NAD^+^ co-crystal and co-crystallisation of His_6_-WbmG with UDP. In both structures the β-phosphate is coordinated by a conserved arginine (Fig. 4a and b), although the precise location of this phosphate is different in the two structures and both are inconsistent with the β-phosphate positions of bound substrate in the structure of a D128N, E129Q mutant of dTDP-glucose 4,6-dehydratase, DesIV, from *S. venezuelae* (Fig. 4c).^23^ In this DesIV structure [Protein Data Bank (PDB) ID 1R6D] the position of the substrate is consistent with the necessary overlap of molecular orbitals required for hydride transfer from the glucose C-4 to the cofactor. It is likely, therefore, that the β-phosphate positions in WbmF and WbmG structures do not represent the positions of equivalent phosphates when the native substrates for these enzymes are bound in the active site.

**Fig. 5:**
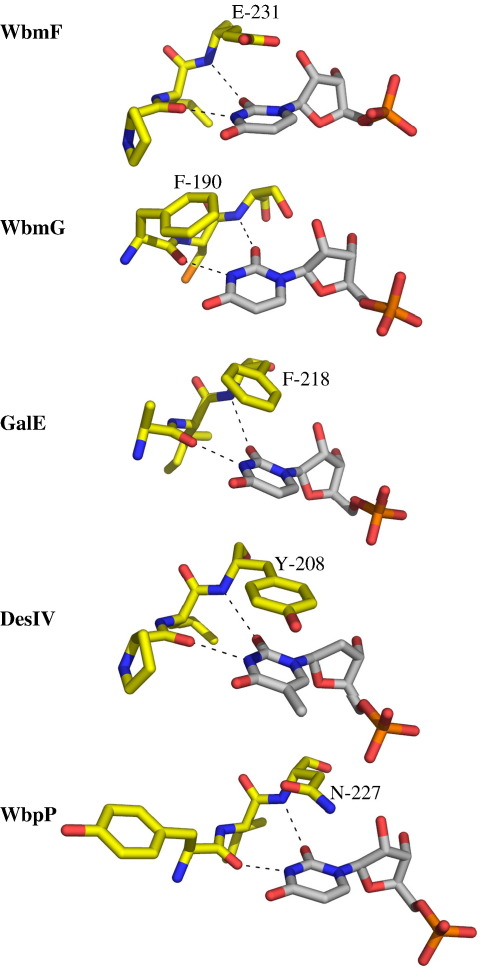
Pi-stacking interactions in binding of uracil or thymine. Amino acids that directly interact with the substrate nucleobase are shown for *B. bronchiseptica* WbmF and WbmG, *E. coli* GalE, *S. venezuelae* DesIV and *P. aeruginosa* WbpP with carbon atoms coloured yellow. UMP or dTDP from each structure is shown with carbon atoms in white. Amino acid side chains that have pi-stacking interactions with the base are labelled, and hydrogen-bonding interactions with the peptide backbone are indicated as dashed lines. Images for GalE, DesIV and WbpP were prepared using PDB files 1LRL,^24^1R6D^23^ and 1SB8,^25^ respectively.

One common feature of substrate binding in SDR enzymes is pi stacking of the nucleobase with an amino acid side chain. In WbmF, this interaction involves Glu231 (Fig. 3a). Unusually, in WbmG the pi-stacking residue (Phe190) is the one that hydrogen bonds with the uracil N-3 (Fig. 3b). In WbmF, *E. coli* UDP-galactose 4-epimerase (GalE),^24^ *S. venezuelae* DesIV,^23^ and *Pseudomonas aeruginosa* WbpP,^25^ the hydrophobic contact is provided by the residue in the *n* + 2 position, whose backbone amide hydrogen bonds with the base's O^2^ group (Fig. 5). This unusual feature of the substrate binding in WbmG could not have been predicted from sequence alignments alone.

### Potential catalytic chemistry

**Fig. 6:**
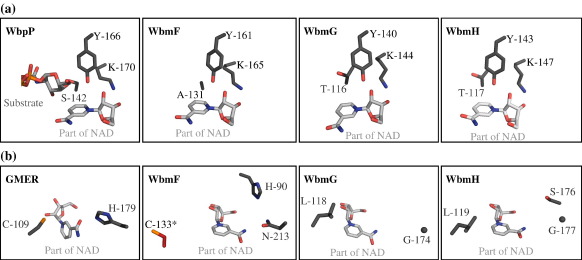
Comparison of active-site residues in WbmF, WbmG and WbmH compared with WbpP^25^ and GMER. (a) The SDR catalytic triad is conserved in WbmG and WbmH but not in WbmF where the residue normally found as serine or threonine superimposes onto the position of Ala131. (b) GMER is shown as an example of an SDR that catalyses 3,5-epimerisation of its substrate.^21^ The GMER epimerase catalytic residues Cys109 and His179 superimpose onto hydrophobic residues in WbmG and WbmH. WbmF has side chains in these positions (Cys133 and Asn213) that may be capable of acid–base chemistry. *Cys133 is not resolved in the WbmF crystal; its position here is inferred from the neighbouring residue (Gly132), which is visible in the electron density.

SDR enzymes have a conserved catalytic triad that is involved in their oxidoreductase activity and that is classically composed of a spatially conserved serine, tyrosine and lysine (SYK) triad, for example, Ser142, Tyr166 and Lys170 in WbpP. The first member of this triad is sometimes found as a threonine (TYK),^23^ and the tyrosine can be replaced by a methionine (SMK).^26^ The SDR triad is conserved in WbmG and WbmH as TYK but in WbmF Ala131 superimposes onto the Ser/Thr position (Fig. 6a).

In the context of O-antigen synthesis in *Bordetella* we are interested in the potential of the *wbm* SDR enzymes to catalyse secondary reactions of their substrates beyond the oxidoreductase reactions common to all SDRs. Of particular significance is the identification of a potential 3,5-epimerase. Despite the lack of a structure in which a full sugar nucleotide substrate analogue is bound, the cavity into which the substrate hexose binds is easily identifiable by comparison with homologue structures and because it is necessarily interposed between the UMP-binding site and the cofactor nicotinamide ring. The active sites of WbmG and WbmH are devoid of amino acid side chains capable of acid–base chemistry except for their respective TYK triads and, in WbmH, Ser176. SDR enzymes that catalyse 3,5-epimerisation of their substrates have a spatially conserved catalytic cysteine and a basic side chain. In GMER these residues are Cys109 and His179;^21^ in GDP-mannose 3,5-epimerase (GME) they are Cys145 and Lys217.^12^ In WbmG and WbmH these amino acids superimpose onto hydrophobic side chains, but in WbmF this catalytic cysteine is conserved (Cys133) and there are two candidates for the basic side chain, Asn213 and His90 (Fig. 6b). Cys133 is disordered in all of our WbmF crystals, indicating that an element of induced fit may be required to bring all of the active-site components into place. His90 has a very unusual backbone conformation, with a *cis* peptide bond to the following residue. This is strongly suggestive of a functional role for this residue, since such conformations are rarely observed except where they are required to place a key side chain in the active site.

### Modelling of the putative substrate into the WbmF active site

**Fig. 7:**
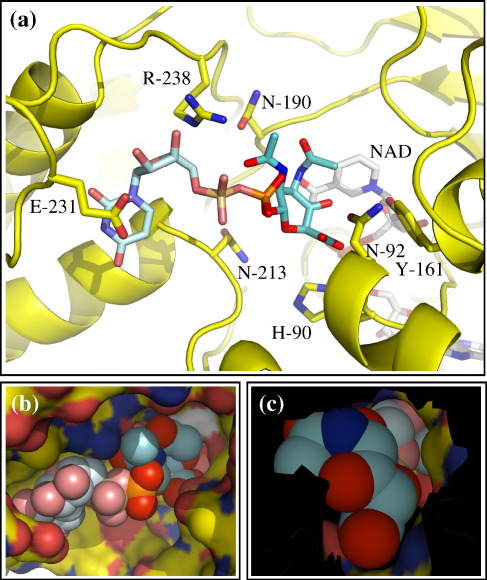
A model of the proposed substrate in the binding site of WbmF. (a) Overview of modelled interaction of WbmF with the 4-keto derivative of UDP-d-ManNAc3NAcA. Protein is shown as cartoon, with key (labelled) side chains, NAD and sugar nucleotide in sticks. The elements of the UDP-sugar found in the experimental structure have paler colours to highlight the modelled sections. (b) and (c) Close-up of the modelled sugar ring (shown as spheres) demonstrates that there are no clashes between the proposed ligand and the protein surface. The two views show opposite sides of the sugar: (c) shows the surface with the imaged slab cut at the level of the first sugar atom, with NAD surface also removed for clarity. Carbon atoms are yellow for protein, white for the NAD and cyan for 4-keto UDP-d-ManNAc3NAcA; oxygen is red, nitrogen is blue, phosphorus is orange and sulfur is purple.

The analysis of the potential catalytic chemistry of WbmF suggested that its role in O-antigen biosynthesis may be to catalyse the 3,5-epimerisation required in the overall conversion of UDP-d-ManNAc3NAcA to UDP-l-GalNAc3NAcA. The 4-keto derivative of UDP-d-ManNAc3NAcA is therefore a likely substrate or reaction intermediate for WbmF. This compound contains a bulkier sugar than any of the substrates of the characterised sugar-nucleotide-modifying SDRs. An important test of our proposed pathway is that the enzymes must be able to accommodate these unusually large substrates. We modelled 4-keto UDP-d-ManNAc3NAcA into the active site of WbmF (Fig. 7). This showed that it is feasible for the substrate to occupy the active site of WbmF in a manner that is consistent with the experimentally determined binding site for UMP, the proximity of the most likely catalytic site, and good geometry.

## Discussion

The abrogation or reduction of O-antigen expression as a result of *wbmF*, *wbmG* or *wbmH* mutations implicates all three genes in the biosynthesis of this molecule, and is consistent with the hypothesis that these three adjacent SDR genes catalyse the UDP-d-ManNAc3NAcA to UDP-l-GalNAc3NAcA conversion, which is probably required for O-antigen synthesis.

Structural analysis of these three *wbm* SDR gene products also enables us to distinguish between them in terms of potential catalytic chemistry. Both WbmG and WbmH have the conserved SDR catalytic triad (TYK in these cases), which suggests that they should be competent to function as oxidoreductases. In both of these enzymes, the pocket that surrounds the active site is hydrophobic and featureless compared with other SDR enzymes; the lack of side chains capable of acid–base chemistry indicates that the functions of these enzymes are likely to be limited to oxidation and/or reduction and probably rules out specific 3,5-epimerase activity. The hydroxyl of Ser176 in WbmH may be capable of facilitating proton exchange; however, an amino acid pair capable of acid–base chemistry is required for 3,5-epimerase function.

Some SDRs that catalyse both oxidation and reduction bind cofactor irreversibly and recycle it within the active site.^27^ In other SDRs, this binding is reversible and the cofactor-binding site is situated at the bottom of a solvent-accessible groove (e.g., in *E. coli* GMER, PDB ID 1BWS).^28^ No such groove is visible in the WbmF, WbmG and WbmH structures, where the cofactor-binding sites are almost completely occluded. However, we do not take this observation as being diagnostic of irreversible cofactor binding in these proteins for the following reasons. Firstly, exogenous cofactor is required for WbmF to be stable in solution with respect to precipitation.^20^ Presumably either cofactor that copurifies with WbmF can be released, resulting in unfolding and aggregation of the protein, or apo-WbmF must bind cofactor supplied from the medium in order to fold correctly. Either case would suggest that NAD can be exchanged between WbmF and solvent. Secondly, human GalE reversibly binds cofactor,^29^ and yet does not have an open binding site like that of GMER. Finally, a conversion of configuration from d-*manno* to l-*galacto* can be catalysed by a single tight NAD^+^-binding enzyme such as GME,^12^ but we cannot envisage a pathway in which two or three such enzymes contribute to this transformation. Instead, we suggest that the protein loops that cover the NAD-binding site may be flexible and move to allow release and replacement of spent cofactor.

WbmF lacks the serine/threonine of the SDR catalytic triad. The conservation of the classical SDR catalytic triad is not absolute amongst members of the family. For example, in the very unusual SDR enzyme, biliverdin IXβ reductase (which catalyses the reduction of a range of substrates including some flavins and non-α-isomers of biliverdin), a histidine occupies the usual place of the catalytic lysine and tyrosine residues.^30^ The shapes and electron delocalisation exhibited by biliverdin IXβ reductase substrates imply that compared with the putative substrates of the Wbm SDR enzymes, they will have rather special requirements for binding geometry and reaction intermediate stabilisation during catalysis. In those SDRs that oxidise or reduce sugar nuclotides at the hexose C-4 position, however, the catalytic triad serine/threonine residue is important because it modifies the p*K*_a_ of the C-4 hydroxyl by forming a short hydrogen bond.^23^ Mutation of the triad threonine (Thr134) to alanine in *E. coli* dTDP-Glc 4,6-dehydratase results in a 200-fold drop in *k*_cat_,^31^ whereas mutation of Ser124 to alanine in *E. coli* GalE reduces epimerase activity (*k*_cat_) almost 3000-fold.^32^ The incomplete WbmF catalytic triad raises doubts about the competence of this enzyme as a sugar nucleotide oxidoreductase. It is possible that the function of the missing side chain is provided by another active-site residue; this implies a different substrate-binding geometry and further studies will be required to investigate this possibility. Conversely, if a standard substrate-binding orientation is assumed, the active site of WbmF resembles that of 3,5-epimerases and our structures of this protein identify Cys133 and either Asn213 or His90 as potential mediators of proton exchange in the epimerisation reaction—one group abstracts a proton from the sugar while the other donates a proton to the opposite face.

By modelling the compound 4-keto UDP-d-ManNAc3NAcA into the WbmF structure we have demonstrated that there is space within this active site to accommodate this bulky sugar nucleotide. Other solutions produced in the modelling process demonstrated alternative plausible substrate positions (data not shown). For this reason, we do not suggest that the solution presented here (Fig. 7) represents the true active conformation. While we did not attempt similar modelling experiments with WbmG and WbmH, visual inspection of these structures indicates that they also have spacious active sites compared with other SDRs and can therefore accommodate diacetamido uronic acids in their hexose-binding pockets.

**Fig. 8:**
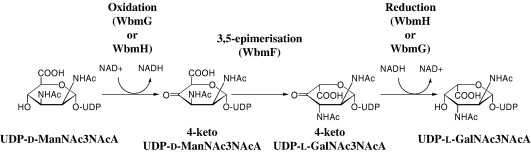
Proposed pathway for the synthesis of UDP-l-GalNAc3NAcA catalysed by WbmF, WbmG and WbmH.

We therefore propose the following biosynthetic pathway (Fig. 8) for the 3,5-epimerisation of UDP-d-ManNAc3NAcA: One of the putative oxidoreductases, WbmG or WbmH, catalyses the initial oxidation at C-4, and the other oxidoreductase is responsible for the final reduction step. Introduction of the keto group lowers the p*K*_a_ of the C-3 and C-5 hydrogen atoms, allowing WbmF to catalyse proton exchange effecting 3,5-epimerisation. This scheme is the most consistent with all of the available data. The function suggested for WbmF is very unusual in that we suggest that this enzyme does not catalyse oxidation or reduction of its substrate. If this is the case, WbmF would be the first such member of the SDR family, and this would imply that the cofactor in WbmF has a purely structural role, without participating in the catalytic cycle. It has been suggested that the transcriptional regulation protein NmrA from *Aspergillus nidulans* is a NAD-binding SDR family member that is not an enzyme at all. NmrA appears to function in control of nitrogen metabolism through physical interaction with the GATA family transcription factor AreA, possibly by controlling the rate of entry of AreA into the nucleus,^33^ and the structural equivalents of the SDR triad tyrosine and serine/threonine in NmrA are Met127 and Met113, respectively.^34^ Since the NmrA structure was reported, however, several SDR enzymes with SMK triads have been reported (e.g., WbpM from *P. aeruginosa*),^26^ so that the nonconservation of the SDR catalytic triad in NmrA should not rule out an enzyme function as absolutely as first appeared.^34^ An alternative possibility to the biosynthetic pathway we have outlined (Fig. 8) is that WbmF does in fact catalyse either oxidation or reduction as well as the 3,5-epimerisation, in which case the third SDR may participate in O-antigen expression by performing a function in the synthesis of the complicated sugar residue that adorns the nonreducing terminus of the O-chain.^35^ The nonredox role we propose for WbmF is consistent, however, with the lack of the conserved SDR triad in this enzyme, and if this protein operates purely as a 3,5-epimerase this may also suggest a mechanism by which the *wbmF* mutant is able to express O antigen, albeit in greatly reduced amounts. The product of the initial oxidation reaction is activated for proton exchange α- to the keto group and it may be that racemisation at these positions occurs at a biologically significant rate without an absolute requirement for enzyme catalysis.

Recent structural studies of the 3,5-epimerase in the dTDP-rhamnose pathway, RmlC,^36^ suggest that the rate-limiting step in its reaction involves a transition state in which the anomeric linkage is in the disfavoured equatorial position (which is also adopted by the reaction product), and that in order to avoid steric clashes and satisfy the requirement for axial proton abstraction, the reaction mechanism requires a twist-boat conformation such as that proposed in the GME reaction.^12^ Binding of substrate in a C-1 equatorial conformation may be part of the way that RmlC reduces kinetic barriers to this reaction. For the same reasons, the product of the proposed WbmF-catalysed 3,5-epimerisation will have the nucleotide attached to the l-GalNAcNAcA in the equatorial position in order to avoid 1,3,5-triaxial clashes. This transformation therefore involves considerable kinetic and thermodynamic barriers and will be slow without enzyme catalysis. However, if sufficient starting material is generated by the upstream oxidase, and the 3,5-epimerisation product is efficiently and selectively removed by the downstream reductase, the flux through this bottleneck may be sufficient to allow O-chain synthesis. Furthermore, the slower 3,5-epimerisation rate resulting from mutation of the *wbmF* gene may make relatively little impact on O-antigen expression levels if this conversion does not usually comprise the rate-limiting step for O-antigen synthesis. A similar effect may operate in the dTDP-l-noviose pathway in *Streptomyces spheroides* where observation of a naturally occurring l-rhamnoside analogue of novobiocin^37^ shows that 3,5-epimerisation of the dTDP-noviose precursor (dTDP-6-deoxy-d-*xylo*-4-hexulose) does occur despite the fact that *in vitro*, the 3,5-epimerisation catalysed by NovW has a 2000-fold lower *k*_cat_ than RmlC^38^ (discussed in Ref. 39).

In conclusion, X-ray crystallography of WbmF, WbmG and WbmH enabled definition of these enzymes' NAD-cofactor preference and the substrate nucleotide specificity. We have been able to distinguish the likely function of WbmF from WbmG and WbmH, but cannot differentiate the roles of WbmG and WbmH. In order to achieve this, these proteins must be characterised *in vitro*. This will require chemical synthesis of UDP-d-ManNAc3NAcA and is beyond the scope of the present study. Subject to future experimental verification of predictions we have made, our data illustrate the usefulness of structural studies for investigating such challenging problems. While they also show why this methodology cannot universally supplant direct *in vitro* characterisation, this kind of structural analysis will prove essential for researchers to make full use of the gene databases.

## Materials and Methods

### Bacterial strains, plasmids and culture conditions

Bacterial strains used in this study are described in Table 3. *Bordetella* was grown on Bordet–Gengou agar (Difco) supplemented with 10% defibrinated horse blood (TCS Cellworks Ltd). *E. coli* was cultured in Luria–Bertani (LB) broth or on LB agar. All strains were incubated at 37 °C and ampicillin (100 μg ml^− 1^), kanamycin (50 μg ml^− 1^), tetracycline (10 μg ml^− 1^ for *E. coli*, 5 μg ml^− 1^ for *B. bronchiseptica*) or streptomycin (200 μg ml^− 1^) were added where required. Suicide plasmids were based on the host-restricted pEX100T backbone^40^ and broad host range shuttle vectors were based on a Km^r^ derivative of pBBR1MCS.^41^ For preparation of LPS, *B. bronchiseptica* was grown in medium supplemented with 50 mM MgSO_4_, as this maximises O-antigen expression in this strain.

### DNA methods

Standard methods were used for DNA manipulations. Oligonucleotides were supplied by Sigma-Genosys. PCR was performed with template from boiled bacteria^42^ and *Taq* DNA polymerase (Promega) or KOD Hot Start DNA polymerase (Novagen).

### Construction of *wbmF*, *wbmG* and *wbmH* mutants

The multiple cloning site (MCS) from pBluescript II SK+ (Stratagene) was excised using SacI and KpnI, blunt-ended and ligated into SmaI-cut pEX100T, to generate the MCS-containing suicide vector pEXMCS. The *wbmF* allelic exchange construct was prepared as follows. The *wbmF* gene in the plasmid Bb540g06 (a clone generated in the *B. bronchiseptica* RB50 genome sequencing project)^43^ was disrupted by insertion of a blunt-ended tetracycline-resistance (*Tc*) cassette into the internal NsiI site. The mutant allele was excised using Acc65I and XbaI, blunt ended, and then cloned into EcoRV-digested pEXMCS. For *wbmG*, the gene was amplified using primers 5′-ATATCTAGACATATGCGTATTCTGATCACCG-3′ (XbaI site underlined) and 5′-ATAAAGCTTTGATTACTGGCAACTCTTC-3′, and the PCR product was topoisomerase cloned into pCR2.1-TOPO using a TOPO-cloning kit (Invitrogen). *wbmG* was excised using XbaI and cloned into the XbaI site in pEXMCS. The *wbmG* gene was then disrupted by cloning a blunt-ended *Tc* cassette into the internal Acc65I site. The *wbmH* mutant allele was obtained by *in vitro* transposon-mediated mutagenesis of the *wbm* locus-containing cosmid, BbLPS1^18^ (accession number AJ007747) using an EZ-Tn5™ <Tet-1> insertion kit (Epicentre). The <Tet-1> transposon, plus flanking *wbmH* DNA, was cut out by partial digestion with AluI and ligated into SmaI-cut pEX100T. Allelic exchange constructs were electroporated into *E. coli* SM10λpir and transferred to *B. bronchiseptica* by conjugation with *E. coli* SM10λpir as donor.^44^ Loss of the plasmid-encoded *sacB* gene in allelic exchange mutagenesis of *B. bronchiseptica* was selected for by growth on LB agar with reduced salt concentration and supplemented with 10% (w/v) sucrose.^45^

### Complementation of mutations

The *B. bronchiseptica* *flaA* promoter was amplified using primers 5′-GCTCTAGATAGGCGCATGCCATGGCC-3′ (XbaI site underlined) and 5′-AAGGATCCCATATGGAGGCTCCCAAGAGAGAA-3′ (BamHI and NdeI sites underlined), and cloned into the XbaI and BamHI sites in pBBR1MCS-kan to generate the vector pCompEmpty. *wbmF* was amplified using primers 5′-AAAAAAACATATGTTGCCAGTAATCATGAATGC-3′ (NdeI site underlined) and 5′-AAAAAGCTTAGATCTAGTCCGCCGTCTTATTTG-3′ (HindIII site underlined) and cloned into pCompEmpty using NdeI and HindIII to generate the *wbmF* complementation vector pCompF. *wbmG* was PCR amplified using primers 5′-AAAAAAACATATGCGTATTCTGATCACC-3′ and 5′-AAAAAGCTTAGATCTGCAACTCTTCAGGTCTTG-3′ and cloned into pCompEmpty using NdeI and HindIII, generating pCompG. *wbmH* was amplified using 5′-GAGAATTCCATATGAAGAAAGTATTTATTACGG-3′ and 5′-AAAAAGCTTAGATCTTTGTCGATGACCAGGATT-3′ and cloned into pCompEmpty using NdeI and HindIII, generating pCompH. Shuttle vectors were moved into *B. bronchiseptica* by conjugation with *E. coli* CC118 as donor^46^ and *trans*-acting transfer functions provided by *E. coli* S17-1 pNJ5000 as helper.^44,47^

### SDS-PAGE, silver stain and immunoblot analysis of LPS

LPS was obtained from *B. bronchiseptica* using a modification of the method of Hitchcock and Brown^48^ as has been described.^49^ SDS-PAGE of LPS was performed using Novex precast 16% tricine gels (Invitrogen). LPS was oxidised in-gel with periodic acid^50^ and visualised with the Silver Stain Plus kit (BioRad). Western blotting was performed as previously reported^51^ using the monoclonal antibody D13B11 that specifically binds the O-antigen-containing LPS of *B. bronchiseptica* strain CN7635E.^35^

### X-ray crystallography

Protein production, purification, crystallisation and data collection were performed as described.^20^ Data were processed using DENZO and SCALEPACK (version 1.97).^52^ Data were truncated and converted to structure factors using TRUNCATE^53^ from the CCP4 Suite.^54^ Molecular replacement was carried out using PHASER^55^ as described.^20^ For the initial WbmG structure, phases were determined experimentally from selenomethionine-labelled protein: peak, inflection point and high-energy remote data were used to determine initial phases using the PHENIX package.^56^ Model building was carried out using COOT,^57^ and refinement was performed with REFMAC version 5.0.^58^ Initial models were improved using ARP/wARP version 6.1 where the data quality permitted.^59^ The CCP4i interface^60^ was used where appropriate. TLS parameters were assigned (where used) using the TLSMD server.^61^ CNS^62^ was used to perform simulated annealing. Structures were validated using COOT, PROCHECK,^63^ WHATCHECK,^64^ and RAMPAGE.^65^ Table 2 provides a summary of the crystallographic statistics. Protein structures were analysed using Pymol,^66^ COOT,^57^ and CCP4MG.^67^ Surface area calculations were performed using the Richards' Rolling Probe method.^68^ More extensive details of the crystallographic methods are provided in the Supplementary Data.

### Modelling of putative substrate into WbmF structure

Coordinates and parameters for the 4-keto derivative of UDP-2,3-dideoxy-2,3-diacetamido-d-mannuronic acid were prepared using the CCP4 monomer library sketcher with refinement in REFMAC 5.2.^58^ An initial structure was prepared by modelling the proposed substrate into the structure of WbmF co-crystallised with UDP in COOT.^57^ The observed density was used to place the atoms present in UMP, and to place sugar atoms in positions occupied by water or glycerol in the observed structure. Different orientations were used in the two molecules in the asymmetric unit to increase the sampling of conformational space. The structure of WbmF co-crystallised with UDP, with 4-keto UDP-d-ManNAc3NAcA placed in, was truncated to include only protein, NAD and 4-keto UDP-d-ManNAc3NAcA atoms. This was used as an input to MODELLER version 9.2 to determine an optimised structure for the complex with the sugar nucleotide.^69^ After modelling, the ligand coordinates were regularised in COOT to correct minor distortions. Ten modelling trials were performed with different seeds, and the best was selected by eye on the basis of geometry, agreement with the determined structures and lack of bad interactions.

### Protein Data Bank accession codes

X-ray amplitudes, phases and the derived atomic coordinates have been deposited with the PDB under the accession codes 2PZJ, 2Q1T, 2Q1U, 2Q1S, 2PZK, 2PZL, 2PZM and 2Q1W.

## Figures and Tables

**Table 1:** Closest homologues of WbmF, WbmG and WbmH in the protein databases

Protein	Closest homologue	Sequence identity (%)	References
WbmF	dTDP-Glc 4,6-dehydratase, RffG, *E. coli*	27	19
WbmG	dTDP-Glc 4,6-dehydratase, DesIV, *S. venezuelae*	28	23
WbmH	dTDP-Glc 4,6-dehydratase, DesIV, *S. venezuelae*	28	23

The sequence identity reported between each *Bordetella* protein and its closest homologue is based on the alignments produced by a third-iteration position-specific iterated BLAST.

**Table 2:** X-ray structures refinement data

PDB ID	2PZJ	2Q1T	2Q1U	2Q1S	2PZK	2PZL	2PZM	2Q1W
Crystal	WbmF-NAD^+^	WbmF-NAD^+^-UDP soak	WbmF-NAD^+^-UDP co-crystal	WbmF-NADH	WbmG-GDP-Man soak	WbmG-UDP-glucose soak	WbmG-UDP co-crystal	WbmH native
Ligands	NAD^+^	NAD^+^, UDP	NAD^+^, UMP	NADH	NAD	NAD, UMP	NAD, UDP	NAD
*Refinement*								
Resolution (Å)	40–1.90	30–1.75	50–1.70	50–1.50	30–2.10	30–2.40	30–2.00	80–2.20
No. unique reflections	27,275	33,895	81,311	57,271	38,661	27,549	51,158	49,975
No. reflections used in refinement	25,919	32,210^a^	77,250^a^	54,327	36,601^a^	26,162	48,524^a^	47,385
*R*_work_/*R*_free_	16.1/20.4	18.3/21.9	17.7/21.1	19.1/22.6	18.8/24.2	18.6/23.5	17.1/22.1	17.9/22.7
No. atoms	2774	2835	5791	2887	5048	4954	5359	7267
Protein	2538	2557	5154	2596	4643	4670	4750	6820
Ligand/ion	44	69	142	44	89	130	136	132
Water	192	209	495	247	316	154	473	415
*B*-factors								
Protein	30.5	41.3	19.7	44.9	34.3	34.6	27.2	29.1
Ligand/ion	21.2	35.6	27.8	27.4	27.5	27.8	39.7	24.4
Water	36.9	40.3	38.2	45.0	38.8	33.3	39.4	31.8
RMSD								
Bond lengths (Å)	0.014	0.015	0.015	0.014	0.014	0.015	0.017	0.016
Bond angles (°)	1.46	1.75	1.54	1.63	1.60	1.59	1.70	1.51
Ramachandran plot^b^								
Favoured (%)	98.1	98.2	98.9	98.8	98.4	97.2	97.9	97.3
Allowed (%)	1.5	1.5	1.1	0.9	1.1	2.1	1.6	2.4
Outliers (%)	0.3	0.3	0.0	0.3	0.5	0.7	0.5	0.3

Data collection statistics have been previously described.^20^*R* = ∑<*F*_obs_ − *F*_calc_>/∑<*F*_obs_>.

a The free set of reflections was included in the final round of refinement.

b Ramachandran plot analysis was performed using RAMPAGE (http://mordred.bioc.cam.ac.uk/~rapper/rampage.php) for which expected values for well-refined, high-resolution structures are 98% favoured, 2% allowed.^65^

**Table 3:** Bacterial strains

Strain	Description or genotype	Reference
*E. coli* SM10λpir	*thi thr leu tonA lacY supE recA∷RP4-2-Tc∷Mu kan*^*r*^*λpir*	44
*E. coli* CC118	*ara*D139, Δ(*ara*, *leu*)7697, Δ*lac*X74, *pho*AΔ20, *gal*E, *gal*K, *thi*, *rps*E, *rpo*B, *arg*E_*am*_, *rec*A1	46
*E. coli* S17-1 pNJ5000	RP4 Res^−^, *tra*^+^, Tc^r^, *pri, Pst*IC^*−*^	44,47
*B. bronchiseptica* CN7635E	Wild type, Sm^r^	51
*B. bronchiseptica* CNF0a	*wbmF∷Tc*^*r*^, Sm^r^	This study
*B. bronchiseptica* CNG1a	*wbmG∷Tc*^*r*^, Sm^r^	This study
*B. bronchiseptica* CNH1d	*wbmH∷Tc*^*r*^, Sm^r^	This study
