# Supplementary material for: Predicting Protein Function from Structure—The Roles of Short-chain Dehydrogenase/Reductase Enzymes in Bordetella O-antigen Biosynthesis
Source: J Mol Biol. 2007 Nov 30;374(3):749–63. doi: 10.1016/j.jmb.2007.09.055 (PMC2279256; doi:10.1016/j.jmb.2007.09.055)
Supplement: Supplementary Data [file mmc1.doc]

SUPPLEMENTARY MATERIAL

**Supplementary figure 1**

**Supplementary figure 1.** Proposed pathway for the biosynthesis of sugar nucleotides required for the assembly of O antigen-containing LPS in *B. bronchiseptica* and *B. parapertussis.* Sugars which are expressed as part of LPS in these organisms are coloured blue. The *wlb*-encoded portion of this pathway is based upon proposed assignments for homologues of the Wlb proteins from *P. aeruginosa*,^70^ which is supported by preliminary characterisation of WlbA and WlbC.^71^ The remaining steps are hypothesised on the basis of bioinformatic and mutational analyses of *wbm* genes.^72^ The 2-epimerase reaction catalysed by WlbD is the only step in this scheme which has been experimentally verified.^11^

**Supplementary figure 2**

**Supplementary figure 2. Examples of electron density modelled as cofactors and nucleotides in these structures.** 2Fo-Fc maps of: (a) electron density modelled as NAD^+^ in the WbmF-UDP soak structure**.** The net represents the contour at approximately 1.5 sigma. (b) density modelled as NADH in the WbmF, NADH co-crystal, contours represent 2 sigma. (c) density modelled as UMP in the WbmF UDP co crystal, at 1 sigma. (d) density modelled as UDP in the WbmF, UDP soak, at 1 sigma. (e) density modelled as NAD^+^ in the native WbmG crystal, at 2 sigma. (f) electron density modelled as UMP in the WbmG-UDP-glucose soak, at 1 sigma. (g) density modelled as UDP in the WbmG, UDP co-crystal, at 1 sigma. (h) density modelled as NAD^+^ in the native WbmH structure, at 2 sigma.

EXTENSIVE DESCRIPTION OF CRYSTALLOGRAPHIC METHODS

Data were collected for all crystals as previously described.^20^ Data were processed using DENZO and SCALEPACK (version 1.97),^52^ and converted to structure factors using TRUNCATE^53^ from the CCP4 suite^54^ (except in the case of data for MAD phasing). For all solutions, model building was carried out using COOT,^57^ and refinement was performed with REFMAC version 5.0.^58^ The CCP4i interface^60^ was used where appropriate. CNS^62^ was used at appropriate intervals to perform simulated annealing when necessary to overcome bottlenecks. Structures were validated using COOT, PROCHECK,^63^ and WHATCHECK.^64^

**Structure solution of WbmF and complexes with substrates**

The structure of WbmF was solved by molecular replacement to the three nearest homologues with solved structures, the dTDP-glucose-4,6-dehydratases (RmlB) from *E. coli*, *Salmonella typhimurium*, and *Streptomyces venezuelae* (PDB codes **1BXK**, **1KEU** and **1R66**). These have sequence identities of 23-25% to WbmF. An initial molecular replacement trial using PHASER^55^ returned one solution that was significantly more likely than other solutions. This solution was corroborated by MOLREP.^73^ Initial maps suggested that the substrate nucleotide binding domain was unlikely to be in a similar conformation to the models, and so new molecular replacement models were built based only on the Rossmann fold domain, with non-homologous side chains cropped to C_γ_, and variant loops removed. These models corroborated the original solution.

The initial phases were improved by density modification using DM,^74^ following which the structure was rebuilt manually. When the quality of the structure had improved sufficiently, ARP/wARP^59^ was used to improve the phases, and conventional methods were used to complete the structure. The final model contained one molecule of WbmF (amino acids 8 to 132, 147 to 301, and 306 to 355), one molecule of NAD^+^, and 192 water molecules.

The structures of WbmF soaked with UDP, or co-crystallised with NADH, were solved using this structure as a starting point. The refined structure was reduced to the Rossman fold domain, with side chains included to C_γ_, to minimise model bias from the lower resolution structure. The structure was rebuilt using ARP/wARP, and then modelled and refined conventionally. The final model of the UDP soak contained one molecule of WbmF (amino acids 6 to 132, 147 to 300, and 305 to 355), one molecule of NAD^+^, one molecule of UDP, and 209 water molecules. The final model of the NADH co-crystal contained one molecule of WbmF (amino acids 6 to 132, 147 to 301, and 303 to 356), one molecule of NADH, and 247 water molecules.

A co-crystal of WbmF with NAD^+^ and UDP crystallised from similar conditions in a different crystal form. Initial phases were provided from molecular replacement using PHASER. The initial model used was the refined structure of WbmF, using the crystallographic dimer around the C2 axis as the model. The molecular replacement solution was then treated in a similar manner to the UDP soak and NADH co-crystals as described above. UDP could be modelled only to the alpha phosphate, and so the final model contains UMP (a likely contaminant of commercial UDP). The final model contained two molecules of WbmF (amino acids 6 to 132 and 147 to 355 of chain A, and amino acids 7 to 132 and 147 to 355 of chain B), two molecules of NAD^+^, two molecules of UMP, two molecules of glycerol, and 495 water molecules.

**Structure solution of WbmG and complexes with substrates**

The initial structure of WbmG was solved using the MAD method, from data collected on a crystal of selenomethionine labelled WbmG. The selenium sites were detected and refined using elements of the *PHENIX* package,^56^ and this package was used to generate an initial model for refinement. As this crystal was nearly isomorphous to the higher resolution native data (complex with GDP-mannose), the remainder of the model was built using the native data. The final model consisted of two molecules of WbmG (amino acids –1 to 308 of chain A, and –6 to 244 and 248 to 306 of chain B), two molecules of NAD^+^, one Mg^2+^ ion, and 316 water molecules. There was no evidence of density for GDP-mannose.

The previously refined model was used as a starting point for refinement of the data for WbmG soaked with UDP-glucose. Significant additional density was apparent, into which the uridine nucleoside, ribose, and one phosphate from UDP-glucose were modelled. There was not sufficient density to model the second phosphate or glucose, and so the final structure was modelled as UMP (a likely contaminant of our commercial source of UDP-glucose). The final model consisted of two molecules of WbmG (amino acids –1 to 308 of chain A, and –6 to 244 and 249 to 306 of chain B), two molecules of NAD^+^, two molecules of UMP, and 140 water molecules.

A co-crystal of WbmG with UDP was obtained in a different lattice. The Rossman fold domain of the refined WbmG structure was used as a model for molecular replacement. PHASER returned a single solution that appeared convincing, as evidenced by strong density for expected features of the structure that were not included in the model. The structure was further refined with ARP/wARP, and then modelled conventionally. The entire UDP molecule could be modelled in this structure. The final model consisted of two molecules of WbmG (amino acids –6 to 309 of chain A and –6 to 306 of chain B), two molecules of NAD^+^, two molecules of UMP, three sulphate molecules, and 473 water molecules.

**Structure solution of WbmH**

WbmH was solved by molecular replacement using PHASER as previously described.^20^ WbmH has 40% sequence identity to WbmG, so the refined structure of WbmG was used as a model for molecular replacement. Three molecules of WbmH were found in the asymmetric unit. It became apparent early in the refinement that the nucleotide-binding domain of WbmH had a different conformation, relative to the Rossmann fold domain, compared to WbmG. It was therefore necessary to rebuild the nucleotide binding domain manually. The final refined structure consists of three molecules of WbmH (amino acids 1 to 257 and 260 to 300 of chain A, 0 to 174, 176 to 258, and 262 to 300 of chain B, and 0 to 299 of chain C), three molecules of NAD^+^, and 415 water molecules.

ADDITIONAL REFERENCES

70. Wenzel, C. Q., Daniels, C., Keates, R. A., Brewer, D. & Lam, J. S. (2005). Evidence that WbpD is an *N*-acetyltransferase belonging to the hexapeptide acyltransferase superfamily and an important protein for O-antigen biosynthesis in *Pseudomonas aeruginosa* PAO1. *Mol Microbiol* **57**, 1288-303.

71. Wing, C. (2004). Biochemical characterisation of lipopolysaccharide biosynthetic enzymes in *Bordetella pertussis* and *Escherichia coli*. PhD Thesis, University of East Anglia, UK.

72. King, J. D. (2006). Characterisation of polysaccharide biosynthesis genes in *Bordetella bronchiseptica*. PhD thesis, University of Cambridge, UK.

73. Vagin, A. & Teplyakov, A. (1997). MOLREP: an Automated Program for Molecular Replacement. *Journal of Applied Crystallography* **30**, 1022-1025.

74. Cowtan, K. (1994). *Joint CCP4 and ESF-EACBM Newsletter on Protein Crystallography* **31**, 34-38.
